# Reduced TCR‐dependent activation through citrullination of a T‐cell epitope enhances Th17 development by disruption of the STAT3/5 balance

**DOI:** 10.1002/eji.201546217

**Published:** 2016-07-12

**Authors:** Christopher Tibbitt, Jane Falconer, Jeroen Stoop, Willem van Eden, John H. Robinson, Catharien M.U. Hilkens

**Affiliations:** ^1^ Musculoskeletal Research GroupInstitute of Cellular MedicineNewcastle UniversityU.K.; ^2^ Rheumatology Research GroupSchool of Immunity and InfectionUniversity of BirminghamU.K.; ^3^ Arthritis Research UK Rheumatoid Arthritis Pathogenesis Centre of Excellence (RACE)U.K.; ^4^ Department of RheumatologyLeiden UniversityThe Netherlands; ^5^ Institute of Infectious Diseases and ImmunologyUtrecht UniversityThe Netherlands

**Keywords:** Citrullination, Cellular activation, STAT3, STAT5, TCR, Th17 cells, T helper cells

## Abstract

Citrullination is a post‐translational modification of arginine that commonly occurs in inflammatory tissues. Because T‐cell receptor (TCR) signal quantity and quality can regulate T‐cell differentiation, citrullination within a T‐cell epitope has potential implications for T‐cell effector function. Here, we investigated how citrullination of an immunedominant T‐cell epitope affected Th17 development. Murine naïve CD4^+^ T cells with a transgenic TCR recognising p89‐103 of the G1 domain of aggrecan (agg) were co‐cultured with syngeneic bone marrow‐derived dendritic cells (BMDC) presenting the native or citrullinated peptides. In the presence of pro‐Th17 cytokines, the peptide citrullinated on residue 93 (R93Cit) significantly enhanced Th17 development whilst impairing the Th2 response, compared to the native peptide. T cells responding to R93Cit produced less IL‐2, expressed lower levels of the IL‐2 receptor subunit CD25, and showed reduced STAT5 phosphorylation, whilst STAT3 activation was unaltered. IL‐2 blockade in native p89‐103‐primed T cells enhanced the phosphorylated STAT3/STAT5 ratio, and concomitantly enhanced Th17 development. Our data illustrate how a post‐translational modification of a TCR contact point may promote Th17 development by altering the balance between STAT5 and STAT3 activation in responding T cells, and provide new insight into how protein citrullination may influence effector Th‐cell development in inflammatory disorders.

## Introduction

Th17 cells are a subset of CD4^+^ T cells defined by production of the signature cytokine IL‐17A (hereafter referred to as IL‐17) 1, 2, 3. Along with IL‐17, these cells are critical producers of IL‐17F, IL‐21, IL‐22 and a source of T‐cell‐derived GM‐CSF 4, 5. These cells provide an important defense against fungal infections and invading extracellular bacteria 6, 7, 8. However, a dysregulated Th17 response can mediate pathogenesis in autoimmune diseases such as psoriasis, multiple sclerosis, and rheumatoid arthritis (RA) 9, 10, 11.

It has been well characterized that the cytokine milieu present during the activation of a naive T cell is important in determining which subset predominates in the adaptive immune response 12, 13, 14. Classically TGF‐β and IL‐6 are thought to induce the generation of Th17 cells, which can be further supported by TNF, IL‐1β, and IL‐23 15, 16. However, this qualitative view of Th responses negates the influence of the T‐cell receptor (TCR) on the fate of precursor cells 17, 18, 19. Seminal studies in the mid‐1990s demonstrated that the Th1‐Th2 balance can be profoundly altered by the density or TCR affinity of the priming peptide 20, 21. Further work has shown a preference for a low density of a high affinity peptide for optimal Treg generation 22. Indeed, our studies have demonstrated that a lower T‐cell activation signal delivered through CD3/CD28 antibodies leads to enhanced generation of human and mouse Th17 cells when compared to a strong CD3/CD28 ‐mediated activation signal 23.

There are over a hundred distinct forms of post‐translational amino acid modifications, which can introduce diversity into proteins 24. Of these the most commonly associated with autoimmune diseases is citrullination, which occurs after the deimination of an arginine to citrulline (Cit) 25. The conversion is catalyzed by a family of calcium‐dependent enzymes termed peptidylarginine‐deiminases (PADIs), of which PADI2 and PADI4 are upregulated in inflammation 26. Citrullinated proteins are more arthritogenic in experimental models of arthritis in rodents 27, 28. In humans, autoantibodies against citrullinated antigens, known as anti‐citrullinated protein antibodies (ACPA), have been shown to be a marker of aggressive arthritis, predictive of erosive disease, and highly specific to RA 29, 30. T cells reacting to citrullinated self‐epitopes releasing pro‐inflammatory cytokines including IL‐17, IL‐6, and/or IFN‐γ have been found in RA patients, suggesting a pathogenic role for these T cells 31, 32, 33.

Here we investigated whether citrullination of a TCR epitope altered Th17 development. To this aim, we used TCR‐transgenic (TCRtg) murine CD4+ T‐cells expressing a TCR specific to an immunodominant peptide derived from the G1 domain of proteoglycan aggrecan (agg), p89‐103. The native form of this peptide contains two arginines, one of which is a putative TCR contact point (R93) 34. We found that the R93‐citrullinated peptide (R93Cit) induced higher levels of Th17‐cell polarization from naïve aggTCRtg CD4^+^ T‐cells than the native p89‐103 peptide, while impairing Th2 development. Furthermore, R93Cit induced lower levels of IL‐2 and CD25 expression, leading to an enhanced ratio of STAT3 to STAT5 phosphorylation, thus linking the intensity of TCR‐dependent T‐cell activation to the pathway critical for Th17 generation. We propose that the degree of TCR‐mediated T‐cell activation influences effector function through controlling the balance between STAT5 and STAT3 activation.

## Results

### Citrullination of the putative TCR contact residue R93 results in a subagonist response

**Figure 1:**
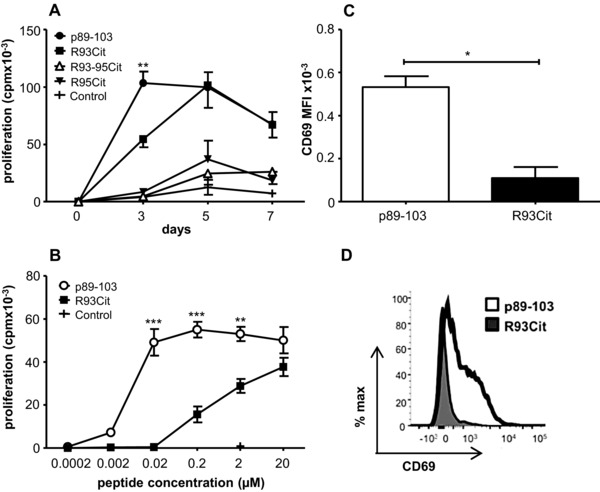
Citrullination of a T‐cell epitope results in reduced T‐cell proliferation. Naïve aggTCRtg T cells (from 5/4E8‐TCR‐Tg BALB/c mice) were co‐cultured with mature syngeneic BMDC together with either native agg peptide p89‐103, peptides citrullinated at either position 93 (R93Cit), 95 (R95Cit) or both (R93‐95Cit), or F1P3 negative control peptide for 3, 5, or 7 days before being pulsed with ^3^H‐Thymidine. (A) Proliferation of aggTCRtg T‐cells. (B) Dose titrations of both p89‐103 and R93Cit; proliferation was measured after 3 days. (C) CD69 expression on aggTCRtg T‐cells after 24 h was determined by flow cytometry. (D) Representative histogram of CD69 expression. Data shown as the mean + standard error of the mean (SEM = SD/√3) of three independent experiments—the mean of three technical replicates from one mouse per experiment; *p*‐values were calculated using two‐way ANOVA; *p<0.05, **p<0.01, ***p<0.001.

We first addressed the question of how citrullination of peptide p89‐103 of the G1 domain of aggrecan affected the magnitude of naïve CD4^+^ T‐cell responses. The native aggrecan peptide contains two arginines offering three potential citrullinated forms (R93Cit, R95Cit, and R93‐95Cit). R93 is an accepted TCR contact point whereas R95 is a putative MHC class II contact point 34. We assessed the ability of LPS‐matured BMDC to induce proliferation of naïve aggTCRtg T cells at a high, fixed peptide concentration (2μM) over a period of 7 days. Citrullination of the putative TCR contact point R93 (R93Cit) resulted in a subagonist response with slower proliferation kinetics as compared to the native peptide p89‐103, although R93Cit‐stimulated T cells displayed similar levels of proliferation as p89‐103‐stimulated T cells at later time points (days 5 and 7; Fig. 1A). In contrast, peptides in which the MHCII contact point R95 had been citrullinated (R95Cit and R93‐95Cit) were not capable of inducing T‐cell expansion above levels of the negative control peptide. To further clarify the differing potential of the native and R93Cit peptide to induce T‐cell proliferation, the dose of both peptides was titrated. After 72h, p89‐103 showed significantly enhanced proliferation when compared to R93Cit over a wide dose range (*p* < 0.01) (Fig. 1B). Moreover, expression of the early activation marker CD69 was also significantly higher in those activated with native p89‐103 than R93Cit (Fig. 1C/D). Thus, the data show that citrullination of the TCR contact residue results in a subagonist response whereas modification of the putative MHCII binding residue completely abrogates the T‐cell response. Therefore, further experiments focused on comparing the native and the R93Cit peptide only.

### Citrullination of the R93 residue favors Th17 cell generation while impairing Th2 development

**Figure 2:**
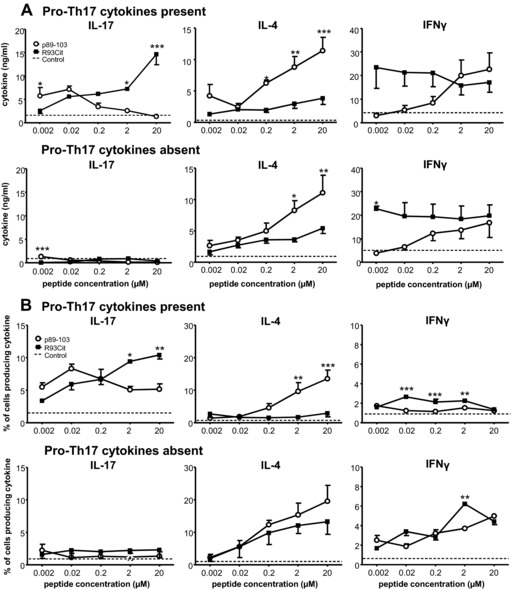
Citrullinated aggrecan peptide favorfavors Th17 cells while impairing Th2 development. Naïve aggTCRtg T‐cells (from 5/4E8‐TCR‐Tg BALB/c mice) were co‐cultured with mature syngeneic BMDC with either p89‐103 or R93Cit in the presence or absence of pro‐Th17 cytokines (IL‐1β/IL‐6/IL‐23/TGFβ). (A) Secondary cultures with p89‐103 and mature BMDC were established before supernatants were collected and IL‐17, IL‐4, and IFN‐γ analyzed by ELISA. (B) Intracellular cytokine production by CD4+ T‐cells was assessed by flow cytometry. The dotted line indicates values obtained from cultures primed with the control peptide F1P3, which is not recognized by the 5/4E8 TCR. Data shown as the mean + standard error of the mean (SEM = SD/√3) of three independent experiments—the mean of three technical replicates from one mouse per experiment; *p*‐values were calculated using two‐way ANOVA; *p<0.05, **p<0.01, ***p<0.001.

Next, we assessed the effect of citrullination of the T‐cell contact residue R93 on the qualitative nature of the CD4^+^ T‐cell response. We used a two‐stage co‐culture system with naïve T cells primed by either native p89‐103 or R93Cit across a range of concentrations in the absence or presence of pro‐Th17 cytokines. After 5 days, when proliferative responses to both peptides had reached the lag phase, cultures were normalized for cell number and re‐stimulated with fresh BMDC and a fixed dose of native peptide; cytokines in supernatants were measured after 48 h. This experimental set up ensured that all secondary cultures received a signal of equivalent strength, allowing comparison of how different T‐cell *priming* conditions affected their polarization. The two peptides led to significant differences in the priming for both IL‐17 and IL‐4 (Fig. 2; for representative flow cytometry plots see Supporting Information Fig. 1). In the presence of pro‐Th17 cytokines, IL‐17 production was promoted at either a low dose of the native peptide or a high dose of the R93Cit peptide (Fig. 2A, upper left panel). These data are consistent with the concept that TCR‐mediated signal strength is regulated by both peptide affinity and density. Notably, IL‐17 production was not supported by either peptide in the absence of pro‐Th17 cytokines (Fig. 2A; lower left panel). In contrast, IL‐4 showed a positive correlation with increasing concentration of native peptide, but with significantly lower levels induced by the citrullinated form in either the absence or presence of pro‐Th17 cytokines (Fig. 2A). IFN‐γ production did not vary significantly over the range of concentrations of R93Cit tested. In contrast, a positive correlation between IFN‐γ production and peptide concentration was observed in those T cells primed with native peptide.

**Figure 3:**
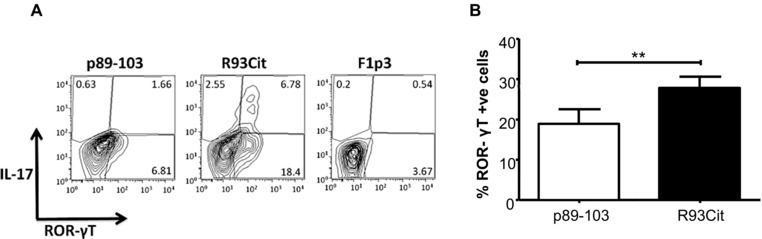
Citrullinated aggrecan peptide enhances ROR‐γT expression. Naïve aggTCRtg T‐cells (from 5/4E8‐TCR‐Tg BALB/c mice) were co‐cultured with mature syngeneic BMDC with either p89‐103 or R93Cit in the presence of pro‐Th17 cytokines (IL‐1β/IL‐6/IL‐23/TGFβ). F1P3 (derived from *Yersinia pestis* capsular protein), an A^d^ binding peptide, was included as a negative control peptide unable to induce proliferation of 5/E4 T cells. ROR‐γT expression and IL‐17 production by CD4^+^ T cells were assessed by flow cytometry. (A) Representative contour plots of three independent experiments with a single mouse per experiment are shown. (B) Percentage of ROR‐γT‐positive cells. Data shown as the mean + standard error of the mean (SEM = SD/√3) of three independent experiments—the mean of three technical replicates from one mouse per experiment; *p*‐values were calculated using two‐way ANOVA; *p<0.05, **p<0.01, ***p<0.001.

We also determined the proportion of cytokine‐producing CD4^+^ T cells by intracellular cytokine staining. A similar picture emerged with the R93Cit peptide inducing a higher percentage of IL‐17^+^ cells and significantly fewer IL‐4^+^ cells at the higher peptide doses, whereas the proportion of IFN‐γ^+^ cells only varied slightly across the peptide dose range and between the two peptides (Fig. 2B). Consistent with the ELISA data, promotion of IL‐17‐producing T cells by a high dose of R93Cit required a pro‐Th17 cytokine environment during T‐cell priming. These observations are supported by a significantly enhanced expression of the Th17‐related transcription factor ROR‐γT induced by the 2μM dose of R93Cit, as compared to native peptide (Fig. 3). Thus, our data demonstrate that while an appropriate cytokine milieu is a contributing factor for Th17 development, a lower TCR‐mediated activation signal at a high peptide density significantly polarizes naïve T cells toward a Th17 phenotype while inhibiting Th2 development.

### Production of other pathogenic Th17 cytokines is enhanced by citrullination of the R93 residue

**Figure 4:**
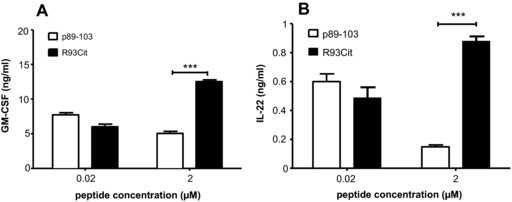
Production of GM‐CSF and IL‐22 is enhanced by peptide citrullination. Naïve aggTCRtg T cells (from 5/4E8‐TCR‐Tg BALB/c mice) were co‐cultured with mature syngeneic BMDC and either p89‐103 or R93Cit in the presence of pro‐Th17 cytokines (IL‐1β/IL‐6/IL‐23/TGFβ). Secondary cultures stimulated with p89‐103 and mature BMDC were established before supernatants were collected and GM‐CSF (A) or IL‐22 (B) measured by ELISA. Data shown as the mean + standard error of the mean (SEM = SD/√3) of three independent experiments—the mean of two technical replicates from one mouse per experiment; *p*‐values were calculated using two‐way ANOVA; *p<0.05, **p<0.01, ***p<0.001.

IL‐17 is an important mediator of inflammation with a role in several chronic diseases, and yet recent studies have shown that it is not the absolute determinant of pathogenicity. Therefore, we sought to assess production of associated cytokines GM‐CSF and IL‐22 known to define Th17 pathogenesis in vivo 35, 36, 37. Naive T‐cell/BMDC co‐cultures were established in the presence of pro‐Th17 cytokines and supernatants of secondary co‐cultures analyzed by ELISA. At the 2 μM dose, the R93Cit peptide promoted significantly more GM‐CSF and IL‐22 production than the native peptide (Fig. 4). We also measured IL‐10, but levels remained below the detection limit of the ELISA (data not shown). Thus, a reduction in TCR‐mediated signal strength through citrullination of a T‐cell epitope can lead to enhanced production of not only IL‐17 but also Th17‐related cytokines.

### Low IL‐2 production in response to R93Cit peptide facilitates the Th17 response

**Figure 5:**
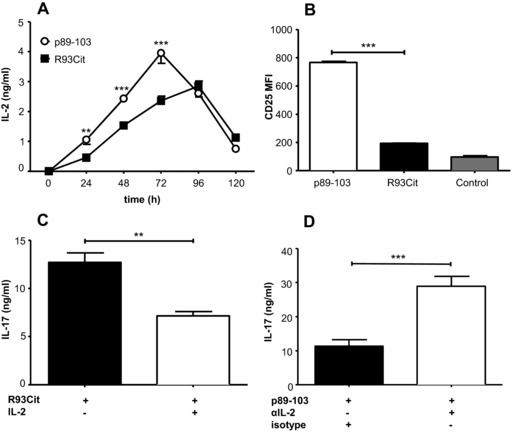
Reduced IL‐2 levels in citrullinated aggrecan peptide stimulated T cells promote Th17 development. Co‐cultures of naïve aggTCRtg T‐cells (from 5/4E8‐TCR‐Tg BALB/c mice) and mature syngeneic BMDC were activated with either p89‐103 or R93Cit in the presence of pro‐Th17 cytokines (IL‐1β/IL‐6/IL‐23/TGFβ). Primary supernatants were collected and (A) IL‐2 levels were measured by ELISA at designated time points and (B) the expression levels of CD25 on CD4^+^ T‐cells after 5 days were determined by flow cytometry. Control: negative control peptide F1P3. The role of IL‐2 in Th17 skewing was assessed by activation of naïve T cells with R93Cit in the presence or absence of rhIL‐2 (C) or p89‐103 in the presence or absence of neutralizing anti‐IL‐2 antibody (D) for 5 days before secondary cultures with p89‐103 and mature BMDC were established. Levels of IL‐17 were analyzed by ELISA. Data shown as the mean + standard error of the mean (SEM = SD/√3) of three independent experiments—the mean of three technical replicates from one mouse per experiment; *p*‐values were calculated using two‐way ANOVA; *p<0.05, **p<0.01, ***p<0.001.

In order to understand the mechanism by which the R93 citrullinated epitope promotes pathogenic Th17 responses, we aimed to assess the role of factors downstream of TCR stimulation. One of the major products of TCR stimulation is IL‐2, known to be a negative regulator of IL‐17 release 38. We therefore sought to define both the levels of IL‐2 and the expression of CD25 (the high affinity component of the IL‐2R) induced by either p89‐103 or R93Cit. IL‐2 production induced by the agonistic p89‐103 peptide was significantly greater during the first 72 h of culture, as compared to levels induced by the subagonist R93Cit peptide (Fig. 5A). The peak release of IL‐2 in response to R93Cit stimulation was also reduced and delayed compared to native p89‐103 stimulation. We also sought to assess the extent to which these T‐cells could respond to IL‐2 and we therefore examined the degree of CD25 expression. Analysis by flow cytometry showed that induction of CD25 by R93Cit was substantially and significantly reduced compared to the native peptide p89‐103 (Fig. 5B; for representative flow plots see Supporting Information Fig. 2).

Given the distinct differences in both IL‐2 production and the level of CD25 receptor expression by native p89‐103‐ and R93Cit‐ stimulated CD4 T‐cells, we wished to clarify whether alterations to the IL‐2 signaling pathway affected Th17 priming. Despite expressing less CD25, addition of rIL‐2 during priming of T cells with R93Cit significantly reduced the production of IL‐17 in secondary cultures (Fig. 5C). Conversely, blocking of endogenous IL‐2 in p89‐103‐stimulated cultures enhanced Th17 development (Fig. 5D). Our data suggest that citrullination of R93 in the epitope leads to a reduction in the IL‐2‐mediated antagonistic effects on Th17 development.

### STAT3:STAT5 ratio is changed in response to the R93Cit peptide

**Figure 6:**
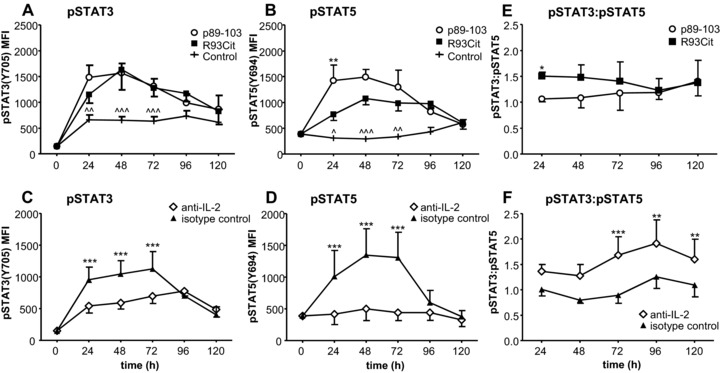
STAT3:STAT5 balance is influenced by peptide citrullination. Naïve aggTCRtg T cells (from 5/4E8‐TCR‐Tg BALB/c mice) were co‐cultured with either p89‐103 or R93Cit and mature syngeneic BMDC in the presence of pro‐Th17 cytokines (IL‐1β/IL‐6/IL‐23/TGFβ). Levels of pSTAT3(Y705) and pSTAT5(Y694) in CD4^+^ T cells were determined by Phosflow after 24, 48, 72, 96, and 120 h. (A, B) MFIs of pSTAT3 and pSTAT5. (C, D), The effect of IL‐2 blockade was assessed using naïve T cells activated with p89‐103 and syngeneic BMDC in the presence of either isotype control or IL‐2 blocking antibody. (E, F) The ratio of pSTAT3:pSTAT5 MFIs was determined for each time point. Data shown as the mean + standard error of the mean (SEM = SD/√3) of three independent experiments—the mean of three technical replicates from one mouse per experiment; *p*‐values were calculated using two‐way ANOVA; *p<0.05, **p<0.01, ***p<0.001. In plots A and B, *indicates significant differences between p89‐103 and R93Cit, and ^indicates significant differences between the control and both p89‐103 and R93Cit.

The balance between STAT3 and STAT5 is thought to be critical in regulating IL‐17 production through competition at the *il17a* locus, with STAT3 promoting and STAT5 inhibiting *il‐17* transcription, respectively 39. Our aim was to assess if the differences in the IL‐2 pathway that we observed between native and R93Cit peptide‐stimulated T cells would be reflected by changes to the pSTAT3:pSTAT5 balance in these cells. To assess the degree of STAT3(Y705) and STAT5(Y694) phosphorylation we activated BMDC/naïve aggTCRtg T‐cell primary cultures with peptides in the presence of pro‐Th17 cytokines and determined phosphorylation of STAT3 and 5 by Phosflow over 5 days (Fig. 6A, B; for representative flow plots see Supporting Information Fig. 3). Interestingly, we found that in both the native and R93Cit peptide‐stimulated cultures similar levels of STAT3 phosphorylation were found, exceeding those found in the control irrelevant peptide‐stimulated cultures (Fig. 6A). Induction of STAT3 phosphorylation could not be attributed to the presence of exogenous pro‐Th17 cytokines IL‐6 and IL‐23 as a similar pattern of STAT3 phosphorylation was observed in T cells stimulated in the absence of these exogenous cytokines, albeit with lower overall levels of STAT3 phosphorylation (data not shown). In contrast, T cells stimulated with p89‐103 gained a higher level of pSTAT5 when compared to R93Cit‐stimulated T cells: this difference was most pronounced and significant at the 24h time point (Fig. 6B). As a result, the pSTAT3:pSTAT5 ratio in R93Cit‐stimulated T cells was significantly higher than in p89‐103‐stimulated T cells at the 24‐h time point (Fig. 6E).

To confirm that the IL‐2 pathway was responsible for the lower pSTAT3:pSTAT5 ratio in p89‐103 stimulated T cells, endogenous IL‐2 was blocked by anti‐IL‐2 antibody in p89‐103‐stimulated DC/naïve T cell cultures. We found that anti‐IL‐2 reduced pSTAT5 activation to background levels (Fig. 6D). A reduction in pSTAT3 was also observed but the downregulation was much less pronounced (Fig. 6C). The balance of pSTAT3 relative to pSTAT5 was shifted in favor of STAT3, leading to a higher pSTAT3:pSTAT5 ratio (Fig. 6F) and explaining why anti‐IL‐2 enhanced the Th17 response (Fig. 5D). In summary, our data are consistent with the notion that the lower affinity of the R93Cit peptide supports lower IL‐2 production and, at early time points, a higher pSTAT3:pSTAT5 ratio, which promotes development of a Th17 phenotype.

## Discussion

Citrullination is a common post‐translational protein modification that occurs during inflammation, but how this affects development of effector T‐cell responses is poorly understood. Here we show that citrullination of the putative TCR contact point (R93) of the arthritogenic aggrecan‐derived peptide p89‐103 has a profound effect on naïve CD4+ T‐cell development. Compared to the native peptide, the R93Cit peptide significantly enhanced the Th17 response, reduced IL‐4‐production, while maintaining support for IFN‐γ secretion at even very low peptide doses. The data suggest that citrullination of the R93 TCR contact point reduces TCR signaling intensity, leading to lower levels of IL‐2 secretion and IL‐2 receptor (CD25) expression. This, in turn, leads to lower levels of STAT5 phosphorylation, thereby reducing the inhibitory action of STAT5 on Th17 development. However, the pro‐Th17 effect of R93Cit was only evident in the presence of pro‐Th17 cytokines, most likely because these cytokines were required for efficient activation of the Th17‐promoting STAT3 pathway. Our data show for the first time how citrullination of a T‐cell epitope can lead to enhanced Th17 differentiation, and also emphasize how the concerted actions of TCR‐ and cytokine‐mediated signaling determine effector T‐cell development.

In this study we chose the agg‐derived p89‐103 peptide because it contains two arginines at two crucial positions: R93 (p2), which is a putative TCR contact point and R95 (p4), a proposed MHCII anchor residue 34, 40. Within polypeptides arginine is a positive charged residue, but citrullination will change the net charge from positive to neutral. Therefore, alterations in the TCR‐ and/or MHCII‐binding capacity of the citrullinated form of the p89‐103 peptide can be expected. Indeed, citrullination of the R93 residue creates a subagonistic peptide in terms of initiating proliferation, IL‐2 production and CD25/69 expression of responding aggTCRtg T‐cells (Figs. 1 and 5). These data are most likely explained by an altered affinity of the R93Cit peptide for the aggTCR, because the binding of this peptide to MHCII (I‐A^d^) has been reported to be similar to binding of the native peptide 41. Citrullination of the R95 residue, on the other hand, resulted in a ‘null‐peptide’ that did not induce any significant T‐cell proliferation above the background (Fig. 1). Therefore, further work focused on the R93Cit peptide.

The regulatory role of TCR signaling has been widely documented with regards to Th1‐ and Th2‐ cell polarization (reviewed in 42), with very high or low levels of TCR activation promoting Th2 responses and intermediate TCR activation levels favoring Th1 cell development. A recent study by van Panhuys *et al*. 19 demonstrated that TCR signal strength controls downstream cytokine receptor expression, thereby influencing the polarizing cytokine cues received by naive Th cells during activation. However, the role of TCR signaling in Th17 cells was not assessed within the remit of that study. We have recently shown that in human memory T cells, a reduced signal through the TCR/CD3 complex promotes Th17 responses 23. We therefore hypothesized that post‐translational modification of T‐cell epitopes leading to changes in TCR signaling strength would affect Th17 polarization of naïve T cells. Our data suggest that citrullination of the putative R93 TCR contact points leads to a decreased signaling strength of the aggTCRtg, as demonstrated by lower proliferation, IL‐2 production, and CD25 expression of responding naïve T‐cells. In agreement with our hypothesis, we found that R93Cit promoted IL‐17‐producing T‐cells, suggesting that lowering the affinity of pMHC–TCR complexes favors Th17 responses.

In addition to the effect of peptide‐affinity on Th17 polarization, we also observed a clear dose‐dependent effect: at higher, saturating peptide doses, R93Cit induced significantly higher levels of IL‐17 than the native peptide; in contrast, at lower, suboptimal peptide concentrations IL‐17 levels were more comparable (Fig. 2). These data are in line with the notion that the overall TCR signaling strength is determined not only by the quality of the MHC‐peptide‐TCR interaction but also by the quantity of the MHC–peptide complexes formed from the relevant peptide epitope. However, peptide affinity and peptide density are mutually exclusive and cannot compensate for one another to produce the same outcome for the T‐cell response, e.g. a high dose of a low affinity peptide does not equate to a low dose of a high affinity peptide or vice versa in terms of downstream TCR signaling. A recent study by Gottschalk *et al*. 43 showed that TCR‐induced gene expression can be broadly segregated into two groups: one that is sensitive to the cumulative TCR signal (dependent on pMHC density) and another that responds to the quality (affinity) of the pMHC complex, independent of pMHC density. In particular, they found that induction of genes involved in the IL‐2 signaling pathway is more dependent on affinity than density of pMHC complexes. Our data also support the notion that peptide density and affinity regulate T‐cell effector development separately. Th17 polarization from naïve T cells was most efficiently promoted by a high density of a low affinity ligand, and lowering the density of a high affinity ligand did not result in a similar level of Th17 polarization.

Our finding that an enhancement of the pSTAT3/pSTAT5 ratio resulted in increased Th17 responses is in line with a previous study showing that STAT5 competes with STAT3 at multiple sites at the IL‐17 locus 39 and negatively regulates its transcription. Thus, the relative activation of STAT3 and STAT5 determines the propensity of naïve T cells to become IL‐17 producers. Our kinetics data suggest that the pSTAT3/pSTAT5 balance early after T‐cell activation (the first 24–48 h) is critically important for Th17 cell development. Indeed, we have previously shown that the level of STAT3 phosphorylation during the first 24–48 h after T‐cell activation plays a critical role in determining human Th17 polarization 23. Our current study is also the first linking the strength of the TCR signal (signal 1) to regulation of Th17 polarization via autocrine IL‐2 signaling.

An alternative way that IL‐2 may regulate Th17 responses was suggested by Liao *et al*. 44; they found that IL‐2 leads to down regulation of IL‐6Ra and gp130 expression, thus interfering with IL‐6‐induced, STAT3‐mediated transcription of critical Th17 genes. While we did not assess IL‐6Ra or gp130 expression directly, STAT3 activation was comparable between both the native and citrullinated forms of aggrecan peptide, inferring signaling by IL‐6 (and IL‐23) was intact in our study.

We observed a sharp decrease in IL‐4‐producing T cells in response to the R93Cit peptide as compared to the native peptide, especially in the presence of pro‐Th17 cytokines (Fig. 2). Moreover, blocking endogenous IL‐2 in native peptide‐stimulated T‐cell cultures completely abrogated the Th2 response (data not shown) The apparent sensitivity of the Th2 response to the strength of TCR signaling fits with the notion that IL‐2‐driven STAT5 signaling is important for IL‐4 expression by creating a permissive chromatin state at the *il4* locus and expression of the transcription factor c‐maf 45, 46, 47. Indeed, some studies have indicated that Th2 express greater levels of CD25 and are dependent on IL‐2 driven STAT5 signaling to initiate early IL‐4 production 48. Thus, these previous studies fit with our observation that the reduced TCR activation strength of the citrullinated peptide blocks Th2 differentiation at an early stage through reduced IL‐2 signaling competency.

A caveat of our study is that the TCR transgenic animal model limits the results to the effects of peptide citrullination on a monoclonal T‐cell population. It is unlikely that citrullination of proteins invariably leads to the promotion of Th17 responses in polyclonal T‐cell populations, representing a diverse T‐cell repertoire where individual T‐cell clones may react differently to citrullinated epitopes. Thus, it can be conceived that different TCRs sense the altered, citrullinated epitope as an agonist, subagonist or even as an antagonist. Indeed, such heterogeneity in the T‐cell response likely underlies the limited effectiveness of clinical trials testing altered peptide ligands in multiple sclerosis patients 49, 50, which was such a promising approach in the corresponding animal model 50, 51. Nevertheless, despite this limitation our study highlights the importance of the strength of TCR‐mediated activation in T‐cell differentiation, and provides a scenario by which a disease‐relevant modification of an immunodominant epitope can alter functional T‐cell development.

IL‐4 has been shown to suppress pathogenesis in inflammatory arthritis models 52, 53; it is, therefore interesting, that T‐cell responses were switched from protective IL‐4 to pathogenic IL‐17/GM‐CSF/IL‐22 as a result of citrullination. It remains to be investigated whether these type of alterations in the Th effector cell response provide an explanation for the previously published observations that the citrullinated form of proteins are more arthritogenic in rodent models 27, 28, and that T cells specific for the citrullinated form of myelin oligodendrocyte glycoprotein exacerbate pathology in the EAE model 54. It should be noted, however, that citrulline reactivity per se is not a prerequisite for developing a Th17 response, as exemplified by psoriasis, a skin disorder characterized by enrichment of Th17 cells but not associated with the presence of ACPA 55, 56. Nevertheless, our data suggest that in a pro‐Th17 environment, citrullination of T‐cell epitopes may further enhance the propensity of T cells to produce Th17 cytokines, including IL‐17 itself but also GM‐CSF and IL‐22. Whether this mechanism is relevant to RA pathogenesis remains to be determined, but the recent observation that ACPA positivity in RA patients is associated with higher proportions of Th17.1 (IL‐17/IFN‐γ‐double producers) and Th22 cells, but not Th1 and Th2 cells 57 provides tantalizing evidence that this is a possible scenario.

In summary, our study demonstrates that citrullination of a T‐cell epitope can enhance Th17 responses by reducing TCR activation strength below that provided by the native epitope. Crucially our results demonstrate a link between TCR activation and the cytokine environment that can alter the response from a Th2‐ to Th17‐dominated response. The balance between IL‐2‐induced STAT5 activation, regulated by TCR signaling, and STAT3 activation, regulated by pro‐Th17 cytokines, is likely to be critical in determining the level of Th17 polarization.

## Materials and methods

### Mice

5/4E8‐TCR‐Tg BALB/c mice are transgenic for a Vβ4/Vα1.1 TCR specific for the immunodominant CD4 T‐cell epitope of human aggrecan, p89‐103 (ATEGRVRVNSAYQDK) 34, 58; where the sequence is numbered 70–84). Transgenic CD4+ T cells are hereafter referred to as aggTCRtg T‐cells. All work was carried out in accordance with the Animals (Scientific Procedures) Act 1986 under the project licence PPL 60/3281 held by JHR. All animals were housed under specific pathogen free (SPF) conditions in Newcastle University's Comparative Biology Centre.

### Peptides

Throughout the study the following synthetic peptides were used (core T‐cell epitope highlighted); p89‐103 (ATE**GRVRVNSAY**QDK), R93Cit (ATE**GCitVRVNSAY**QDK), R95Cit (ATE**GRVCitVNSAY**QDK), R93‐95Cit (ATE**GCitVCitVNSAY**QDK) and the negative control peptide F1p3 (AADLTASTTATATLVEPARI; derived from *Yersinia pestis* capsular protein) not recognized by the 5/4E8 TCR but capable of binding to A^d^. All peptides were purchased from JPT peptide technologies GmbH, Germany, >80% purity.

### Cell isolation and culture

For generation of bone marrow‐derived DC (BMDC), bone marrow was extracted from tibias and femurs of nontransgenic littermates and grown in RPMI‐1640 (Sigma, UK); with 10% FBCS (PAA Laboratories, UK), with 2 mM Glutamine, 100 units/mL penicillin, 100μg/mL of streptomycin and 50μM β‐mercaptoethanol (all Sigma, UK) supplemented with 20 ng/mL of granulocyte‐macrophage colony‐stimulating factor (GM‐CSF; R&D Systems, USA) at 2 × 10^6^ cells/mL in 10 mL in Petri dishes. Medium supplemented with GM‐CSF was refreshed on days 3, 6, 8, and 10 of culture. On day 10, BMDC were matured in 0.1 μg/mL lipopolysaccharide (LPS; Sigma, UK)). After 24 h, cells were harvested using a cell lifter. Naive CD4^+^CD62L^hi^CD44^lo^ T‐cells were isolated by magnetic bead labelling (Miltenyi Biotech, Germany) according to the manufacturer's protocol. Isolations routinely yielded >95% purity.

### BMDC/T‐cell co‐cultures

Unless otherwise stated, 1.25 × 10^5^ isolated naive CD4^+^ AggTCRtg T‐cells were co‐cultured with 6.25 × 10^3^mature BMDC (1:40 ratio of BMDCs: T cells) in IMDM (Sigma, UK); 10% serum replacement (Invitrogen); 100 units/mL penicillin; 100 μg/mL streptomycin; and 50 μM β‐mercaptoethanol. Primary culture was carried out for 5 days with peptide doses ranging from 2 × 10^−4^ μM to 20 μM (2 μM if not stated otherwise) and with or without exogenous Th17‐skewing cytokines; IL‐1β (10 ng/mL; Peprotech, UK), IL‐23 (10 ng/mL; Peprotech, UK), TGF‐β (10 ng/mL; R&D Systems, USA), and IL‐6 (50 ng/mL; Peprotech, UK). Proliferation was assessed by addition of 10 kBq ^3^H‐thymidine (specific activity; 74.0 GBq/mmol; Perkin Elmer, USA) for the last 12 h of culture. Cell DNA was harvested onto fibreglass filter mats and radioactivity quantified by beta scintillation spectroscopy (Perkin Elmer Microbeta TriLux; Perkin Elmer, USA). On day 5 secondary cultures were established using 4 × 10^5^ T cells and 2 × 10^3^ fresh mature BMDC with 2 μM of p89‐103. Supernatants were collected after 48 h and analyzed for cytokine levels by ELISA (see below). Alternatively, cells were stimulated with PMA (50 ng/mL, Sigma) and ionomycin (I) (500 ng/mL, Sigma) for 5 h at 37°C. Brefeldin A (BFA (Sigma, UK) was added for the last 4 h. Intracellular cytokine staining was performed as described under flow cytometry.

### Enzyme‐linked immunosorbant assay (ELISA)

Supernatants from co‐cultures were extracted and frozen at −20°C until required. ELISA was performed for the relevant cytokine according to the manufacturers’ protocols (IL‐17, IL‐4 and IFN‐γ ‐ R&D Systems, USA; IL‐22, and GM‐CSF ‐ eBiosciences, UK).

### Flow cytometry

Cell surface and intracellular phenotypes were determined by flow cytometry using the following reagents: anti‐CD4‐Alexa 488 (0.2 μg/mL, RM4‐5); anti‐ROR‐γT‐PE (2 μg/mL, Q31‐378); anti‐IL‐4‐PE ((2 μg/mL, BVD4‐1D11) BD Biosciences, UK); anti‐CD25‐eFluro450 (0.2 μg/mL, eBio3C7); anti‐IL‐17‐APC (0.2 μg/mL, eBio17B7), anti‐CD69‐PE (0.2 μg/mL, H1.2F3), and anti‐IFN‐γ‐PEcy7 ((0.2 μg/mL, XMG1.2) eBioscience, UK). For intracellular cytokine staining, cells were blocked with 200 μg/mL of rat serum (Sigma, UK) and fixed and permeabilized using Foxp3 staining kit (eBioscience) according to manufacturer's instructions. Data were acquired using FACS Canto‐II (BD) and analyzed by Flowjo software 8.7.1 (Treestar, USA). The following gating strategy was used (see also Supporting Information Fig. 1: (i) a ‘life’ gate was set on the FSC/SSC plot; (ii) a gate was set on CD4+ cells, based on an isotype‐matched control plot; (iii) marker of interest‐positive cells were gated on the basis of a fluorescence‐minus‐one plot – unstained and isotype‐matched control samples were used to exclude nonspecific binding of mAbs used.

### STAT3/STAT5 phosflow

Parallel co‐cultures of naive T cells and BMDC were established with 2 μM of either p89‐103 or R93Cit in the presence of pro‐Th17 cytokines. These were harvested at time points between 0 and 120 h and permeabilized using appropriate buffers (Cytofix Fixation Buffer and Perm Buffer III; BD Bioscience, UK). Cells were incubated with 200 μg/mL of mouse serum (Sigma, UK) for 15 min prior to the addition of antibodies. In addition to anti‐CD4‐Alexa fluor 488, cells were stained using Phosflow antibodies pSTAT3(Y705)‐PE and pSTAT5(Y694)‐PEcy7 (BD Biosciences, UK) for an hour before washing and resuspension in FACS buffer (PBS (Ca^2+^Mg^2+^ free), 0.2% BSA, 0.09%NaN_3_). Data were acquired and analyzed as above.

### Statistical analysis

For comparisons between two groups paired *t*‐tests were used to determine significance. In cases with multiple groups or time points, *p*‐values were determined utilizing two‐way ANOVA analysis. All analyses were determined using Graphpad Prism 5.0 software.

## Conflict of interest

The authors declare no commercial or financial conflict of interest.

**Abbreviations**
ACPA: anti‐citrullinated protein antibodies
agg: aggrecan
BMDC: bone marrow‐derived dendritic cells
Cit: citrulline
PADIs: peptidylarginine‐deiminases
R93Cit: p89‐103 peptide of the G1 domain of aggrecan citrullinated on residue 93
RA: rheumatoid arthritis
SEM: standard error of the mean
TCR: T‐cell receptor, TCRtg, TCR‐transgenic

## Supporting information

As a service to our authors and readers, this journal provides supporting information supplied by the authors. Such materials are peer reviewed and may be re‐organized for online delivery, but are not copy‐edited or typeset. Technical support issues arising from supporting information (other than missing files) should be addressed to the authors.

Peer Review Correspondence

Supporting information
