# Supplementary material for: Reduced TCR‐dependent activation through citrullination of a T‐cell epitope enhances Th17 development by disruption of the STAT3/5 balance
Source: Eur J Immunol. 2016 Jul 12;46(7):1633–43. doi: 10.1002/eji.201546217 (PMC4949576; doi:10.1002/eji.201546217)
Supplement: Supplementary file 2 — Supporting information [file EJI-46-1633-s002.pdf]

European Journal of Immunology

Supporting Information

for

DOI 10.1002/eji.201546217

Christopher Tibbitt, Jane Falconer, Jeroen Stoop, Willem van Eden, John H. Robinson and Catharien M.U. Hilkens

Reduced TCR-dependent activation through citrullination of a T-cell epitope enhances Th17 development by disruption of the STAT3/5 balance

© 2016 The Authors. *European Journal of Immunology* published by WILEY-VCH Verlag GmbH & Co. KGaA, Weinheim.

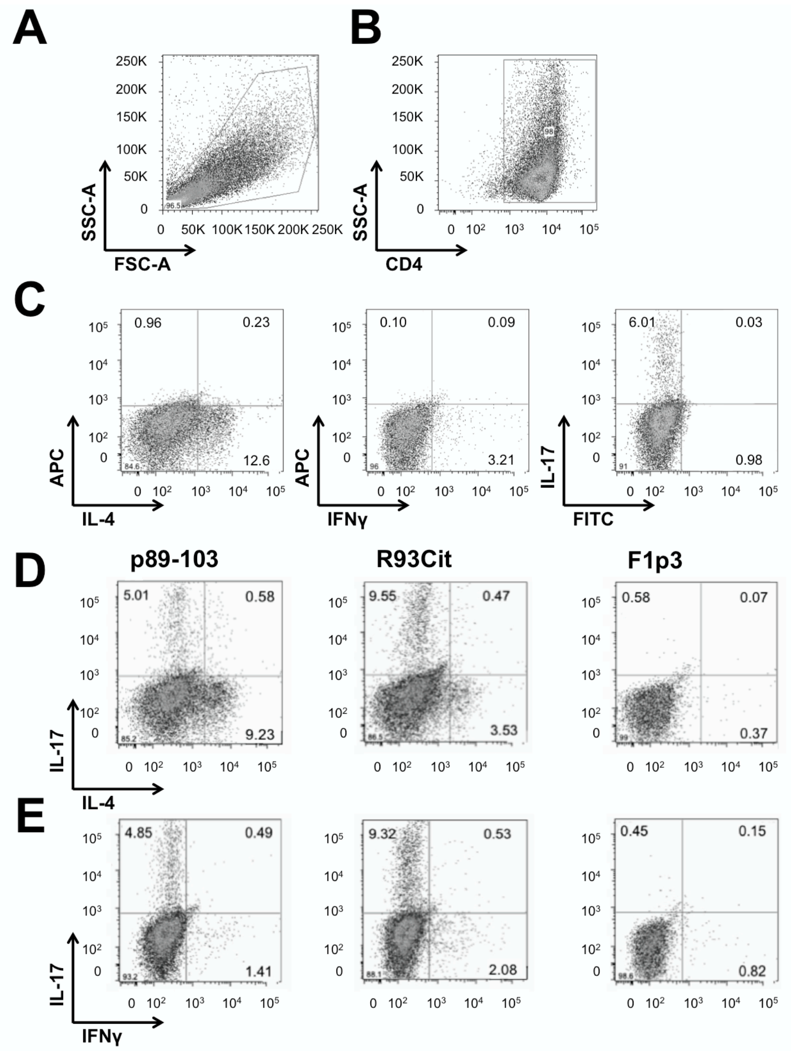

Supporting Information Figure 1. Example of gating strategy used to assess samples using (A) rough live/ dead gate (B) gating on CD4 (C) with cytokines gates set using FMO control to set final gates for each cytokine (IL-4-PE, IFN$_{\gamma}$-FITC and IL-17-APC). Naïve aggTCRtg T-cells (from 5/4E8-TCR-Tg BALB/c mice) were co-cultured with mature syngeneic BMDC for 5 days with pro-Th17 cytokines (IL-1β/IL-6/IL-23/TGFβ) and 2μM of either p89-103, R93Cit or the negative control peptide F1p3. For intracellular cytokine analysis (IL-17, IL-4, IFN-γ), cells were re-stimulated with PMA/I for 1h, brefeldin A was added for a further 5h and cytokine production by CD4+ T-cells was assessed by intracellular labelling and flow cytometry. Representative flow plots of IL-17 against IFN-γ (D) and IL-17 against IL-4 (E) are shown above.

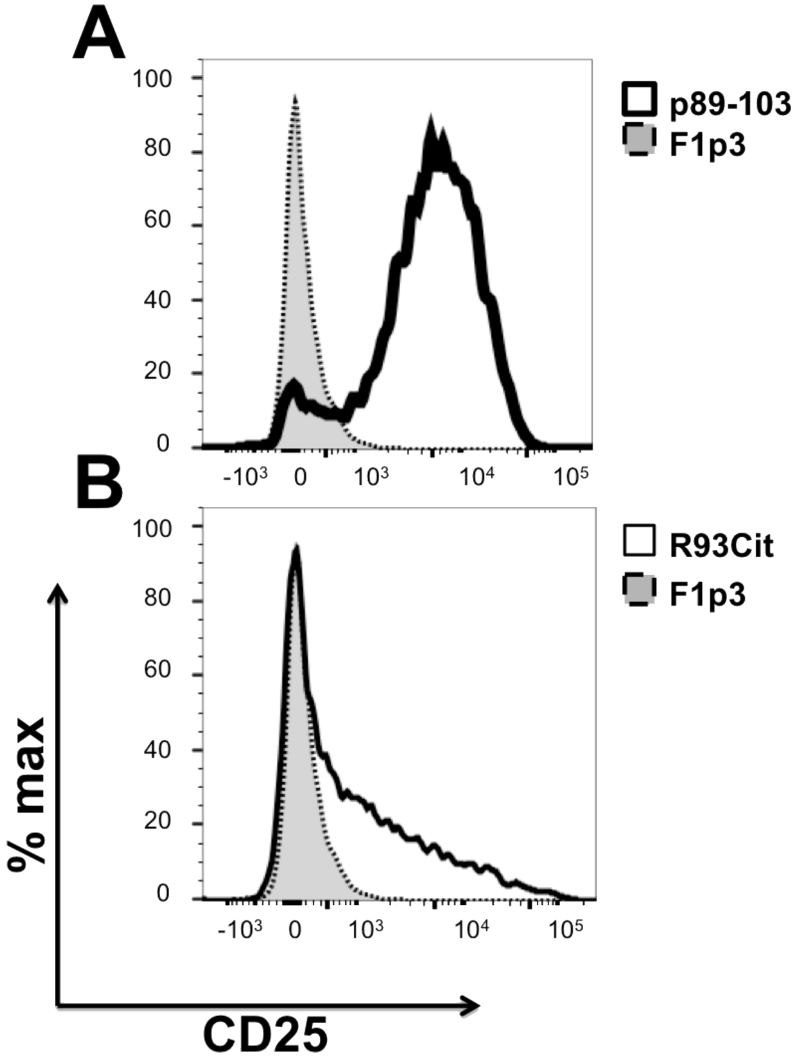

Supporting Information Figure 2. Naïve aggTCRtg T-cells (from 5/4E8-TCR-Tg BALB/c mice) were co-cultured with mature syngeneic BMDC for 5 days with pro-Th17 cytokines (IL-1β/IL-6/IL-23/TGFβ) and 2μM of either p89-103, R93Cit or the negative control peptide F1p3. Cells were harvested and surface expression of CD25 on CD4+ T cells determined by flow cytometry, applying the same gating strategy as shown in Supporting Information Figure 1A, B. Representative histograms are shown for both p89-103 (A) and R93Cit (B) relative to F1p3.

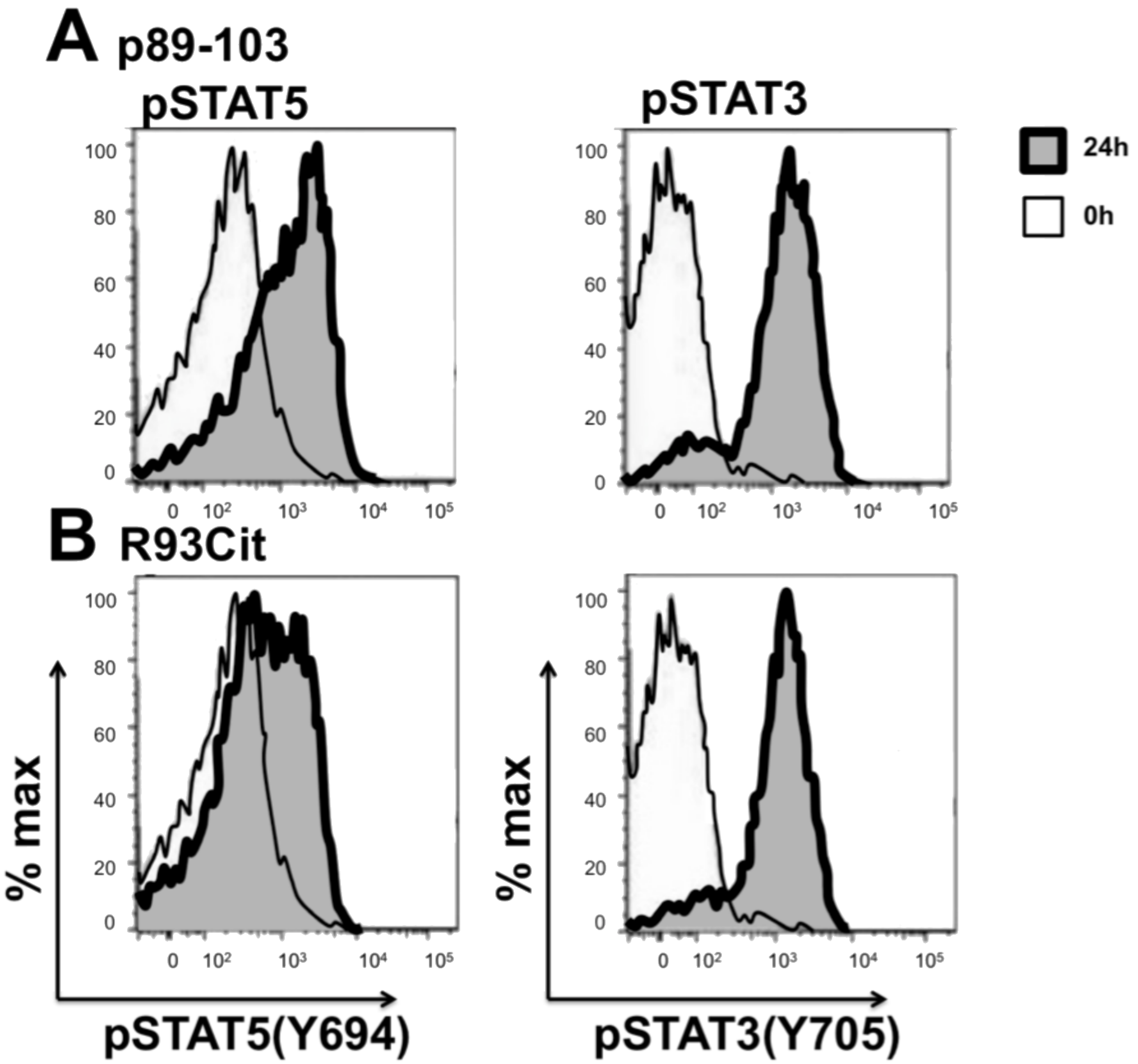

Supporting Information Figure 3. Naïve aggTCRtg T-cells (from 5/4E8-TCR-Tg BALB/c mice) were co-cultured with mature syngeneic BMDC for 5 days with pro-Th17 cytokines (IL-1β/IL-6/IL-23/TGFβ) and 2µM of either p89-103, R93Cit or the negative control peptide F1p3. Cells were harvested and expression of phosphorylated (p)STAT3 and pSTAT5 by CD4+ T-cells was determined by flow cytometry. The same gating strategy as shown in Supporting Information Figure 1A, B was applied. Representative histograms of pSTAT5(Y694) and pSTAT3(Y705) are shown for p89-103 (A) and R93Cit (B) relative to basal pSTAT levels on freshly isolated, unstimulated T-cells.
