# Supplementary material for: Reduced TCR‐dependent activation through citrullination of a T‐cell epitope enhances Th17 development by disruption of the STAT3/5 balance
Source: Eur J Immunol. 2016 Jul 12;46(7):1633–43. doi: 10.1002/eji.201546217 (PMC4949576; doi:10.1002/eji.201546217)
Supplement: Supplementary file 1 — Peer Review Correspondence [file EJI-46-1633-s001.pdf]

European Journal of Immunology

**Reduced TCR-dependent activation through citrullination of a T-cell epitope enhances Th17 development by disruption of the STAT3/5 balance**

Christopher Tibbitt, Jane Falconer; Jeroen Stoop, Willem van Eden, John Robinson, Catharien Hilkens

Correspondence: Dr. Catharien Hilkens, Institute of Cellular Medicine, Musculoskeletal Research Group, Newcastle University, Newcastle upon Tyne, NE2 4HH, United Kingdom

---

| Review Timeline: | Submission date: | 26-Nov-2015 |
|---|---|---|
| | First Editorial decision: | 23-Dec-2015 |
| | Revision/s received: | 22-Mar-2016 |
| | Second Editorial decision: | 15-Apr-2016 |
| | Accepted: | 09-May-2016 |

---

Handling Executive Committee member: Prof. Iain McInnes

Please note that the correspondence below does not include the standard editorial instructions regarding preparation and submission of revised manuscripts, only the scientific revisions requested and addressed.

**First Editorial Decision**
**23-Dec-2015**

Dear Dr. Hilkens,

Manuscript ID eji.201546217 entitled "Reduced TCR-dependent activation through citrullination of a T-cell epitope enhances Th17 development by disruption of the STAT3/5 balance" which you submitted to the European Journal of Immunology has been reviewed.

The comments of the referees are included at the bottom of this letter. A revised version of your manuscript that takes into account the comments of the referees will be reconsidered for publication. Should you disagree with any of the referees concerns, you should address this in your point-by-point response and provide solid scientific reasons for why you will not make the requested changes.

You should also pay close attention to the editorial comments included below. **In particular, please edit

your figure legends to follow Journal standards as outlined in the editorial comments. Failure to do this will result in delays in the re-review process.**

Please note that submitting a revision of your manuscript does not guarantee eventual acceptance, and that your revision will be re-reviewed by the referees before a decision is rendered.

If the revision of the paper is expected to take more than three months, please inform the editorial office. Revisions taking longer than six months may be assessed by new referee(s) to ensure the relevance and timeliness of the data.

Once again, thank you for submitting your manuscript to European Journal of Immunology and we look forward to receiving your revision.

Yours sincerely,
Laura Soto Vazquez

On behalf of
Prof. Iain McInnes

Editorial Office
European Journal of Immunology
e-mail: ejied@wiley.com
www.eji-journal.eu

********************

Reviewer: 1

Comments to the Author

This article by Tibbit et al describes a model system in which the differentiation of TCR-transgenic T-cells is studied when activated with a wild-type peptide ligand or a citrullinated (altered) peptide ligand. The authors show that the citrullinated peptide is recognized less well by TCR-expressing T-cells and that this is associated with an enhanced differentiation towards a Th17-cell phenotype when cells are cultured in the presence of Th17-skewing cytokines. The authors also show that T-cells responding to the citrullinated peptide produced less IL-2, expressed lower levels of the IL-2 receptor subunit CD25, and showed reduced levels of STAT5 phosphorylation, whilst STAT3 activation was unaltered. A similar phenotype

was observed when transgenic TCR-expressing cells where activated with the wild-type peptide in the presence of IL-2 blocking agents. The authors conclude that a post-translational modification of a TCR contact point may promote Th17 development by altering the balance between STAT5 and STAT3 activation in responding T-cells.

Comments:
This is a well-written paper that mimics the studies performed in the 1990s on altered peptide ligands and their effects on T-cell skewing. The novelty of the current manuscript is found, in my opinion, by the finding that citrullinated epitopes can influence T-cell reactivity by changing the outcome of T-cell responses/skewing. Currently, most work performed in the field of T-cell reactivity against citrullinated peptides follows the concept that citrullination creates new T-cell epitopes that can be recognized by T-cells that do not recognize the wild-type sequence. Thus, the manuscript by Tibbit et al presents a new concept that has not been investigated before.

A clear limitation of the studies presented is that only one TCR-used and hence it is not known if this concept also applies for polyclonal T-cell responses. My only specific comment would be that the authors should mention more specifically that it is not known whether the findings presented would also apply for polyclonal T-cell responses but that the studies performed in the 1990s on altered peptide ligands indicate that this is questionable.

Reviewer: 2

Comments to the Author

The aim of the article Reduced TCR-dependent activation intensity through citrullination of a T-cell epitope enhances Th17 development by disruption of the STAT3/5 balance was to identify citrullination as a novel mechanism for 1) reducing TCR-dependent activation 2) enhancing Th17 T cell differentiation.

General concerns:
The study is based on the finding that citrullination of one peptide alters TCR recognition. Based on previous work, R93 has been demonstrated to be a TCR contact point. The authors clearly demonstrate that upon incubation with R93Cit, T cells produce less IL-2 and express less CD25 but donÂ´t provide direct evidence that citrullination modify T-cell recognition. Showing early TCR defects upon incubation with DC loaded with R93Cit would strengthen the study. Read-out could include calcium flux analysis or early phosphorylation markers. Additional early surface markers such as CD69or CD44 could also be included.

Moreover, the discussion should be extended with regard to the relevance of this study in the setting of a diverse T cell repertoire (top of page 15), when one would expect other T cells to be activated by the citrullinated peptide as compared to the native version. This angle could also impact the conclusion in the abstract.

Lastly, therapeutic intervention of the IL-17 pathways are so far looking more promising in the setting of Psoriasis, Psoriatic Arthritis and Spondylopathies as compared to Rheumatoid Arthritis, and only the latter disease is characterized by autoimmunity to citrullinated peptides and proteins. The author may want to discuss also this aspect when extrapolating their murine studies to a human setting, i.e. why IL-17 interference has not been more successful in the setting of RA.

Detailed questions and comments:

Page 2 line25, abstract
The peptide citrullinated on residue 93 (R93Cit) significantly enhanced Th17 development
The authors have chosen a biased system and should mention that pro-IL-17 cytokines are also necessary.

Page 5 line 26
Response with slower proliferation kinetics as compared to the native peptide p89-103
At day 5 and 7, no difference in proliferation is observed between p89-103 and R93 Cit. Could you comment on that and also why 5 days were used for the following experiments (Figure 2 for example)?

Page 6 line 17
re-stimulated with fresh BMDC and a fixed dose of native peptide•
Could the authors explain why the native peptide was used for re-stimulation?

Figure 1A versus 1 B
In Figure 1 A, at 72 hours, proliferation value for p89-10 is around 100. In Figure 1B, at 72 hours, proliferation value for p89-10 is around 60. Could you comment on that difference?

Figure 2A
Could the control peptide be represented as a dotted line instead of one single point?
Even low concentration of R93Cit induces production of IFNg as compared to p89-103 (with or W/O Pro-Th17 cytokines). Please comment and discuss this aspect in your manuscript? Specially I would moderate the sentence in discussion page 11 line 21• In contrast, the IFN-Î³ response was only

marginally affected

Figure 2B

Please include a representative flow-cytometry plot (dot blot) for each of the cytokine tested (Can be included in supplementary).

Please change the y-axis (% of cytokine producing cells) or include abbreviations in the legend.

no increase in the % of IFNg secreting cells are observed when increasing the peptide concentration of p89-103 as compared to cytokine secretion (Figure 1A) .Could you please comment this technical aspect?

Figure 3A

Could you include description of peptide control F1p3 in the legend?

Figure 3

Can you change the y-axis (% of RORgT producing cells) or explain abbreviation in the legend?

Figure 5B

Please provide a representative histogram plot (supplementary).

Figure 5C. Have you also tested additional pro-Th17 cytokines (GM-CSF and Il-22)?

Figure 6: include a representative histogram plot for pSTAT3 and STAT5 at day 0 and 24 with p89-103 and R93Cit.

**First Revision – authors' response**

**22-Mar-2016**

Point-by-point replies to the reviewers' comments

Reviewer 1

We thank the reviewer for their positive feedback and comments.

European Journal of Immunology

‘A clear limitation of the studies presented is that only one TCR-used and hence it is not known if this concept also applies for polyclonal T-cell responses. My only specific comment would be that the authors should mention more specifically that it is not known whether the findings presented would also apply for polyclonal T-cell responses but that the studies performed in the 1990’s on altered peptide ligands indicate that this is questionable.’

The reviewer raises the valid point that our findings apply to a monoclonal T cell population, but may not apply to a polyclonal T cell population. We would like to stress, however, that questions regarding how a particular TCR (with known fine specificity) responds to changes in the peptide ligand, can only be addressed by using a monoclonal T cell population – otherwise we would not be able to distinguish between one T cell (clone) responding differently to an altered peptide ligand, or different T cells responding to different ligands.
As requested by the reviewer, we now are more explicit in discussing this limitation (page 15). We now clearly state that individual T cell clones in a polyclonal population may react differently to altered (citrullinated) T cell epitopes and that it is unlikely that citrullination of proteins invariably leads to the promotion of Th17 responses in polyclonal T-cell populations.

Reviewer 2

We’d like to thank the reviewer for their constructive feedback and detailed comments.

Response to general comments:

‘The study is based on the finding that citrullination of one peptide alters TCR recognition. Based on previous work, R93 has been demonstrated to be a TCR contact point. The authors clearly demonstrate that upon incubation with R93Cit, T cells produce less IL-2 and express less CD25 but don´t provide direct evidence that citrullination modify T-cell recognition. Showing early TCR defects upon incubation with DC loaded with R93Cit would strengthen the study. Read-out could include calcium flux analysis or early phosphorylation markers. Additional early surface markers such as CD69or CD44 could also be included.’

In response to the reviewer’s comment that showing early TCR defects upon R93Cit recognition would strengthen the study we performed further experiments to measure phosphorylation of the TCR-associated kinase ZAP70 by flow cytometry. We indeed found that ZAP70 phosphorylation in response to R93Cit were impaired in comparison to the native peptide (especially at 20 mins), but the shifts we observed in the flow plots were marginal and we chose not to publish these data (See Figure A at the end of this letter). Indeed, an experimental repeat showed even less convincing shifts than the example shown in Figure A (not shown). However, we also followed the suggestion of the reviewer to look

at the expression of an early T-cell activation marker, CD69, and we have now included these data in revised Figure 1 (data are described on page 5 of the revised manuscript). The data show that the native p89-103 peptide induces a higher level of CD69 expression than the R93Cit peptide, which is in line with our proliferation, IL-2 and CD25 data. We therefore feel that we provide sufficient evidence that the level of T-cell activation in response to R93Cit is reduced (lower CD69, CD25 and IL-2 production) compared to the native peptide.

‘Moreover, the discussion should be extended with regard to the relevance of this study in the setting of a diverse T cell repertoire (top of page 15), when one would expect other T cells to be activated by the citrullinated peptide as compared to the native version. This angle could also impact the conclusion in the abstract.’

We have extended the discussion (page 15) regarding the implication of our study with respect to polyclonal T-cell responses – see also response to the similar comment by reviewer 1. We have also toned down the concluding remark in the Abstract (replaced ‘can’ for ‘may’) indicating that our work only represents a possible scenario. Unfortunately the limited word count in the Abstract does not allow for a clearer detailed discussion of our findings in this section.

‘Lastly, therapeutic intervention of the IL-17 pathways are so far looking more promising in the setting of Psoriasis, Psoriatic Arthritis and Spondylopathies as compared to Rheumatoid Arthritis, and only the latter disease is characterized by autoimmunity to citrullinated peptides and proteins. The author may want to discuss also this aspect when extrapolating their murine studies to a human setting, i.e. why IL-17 interference has not been more successful in the setting of RA.’

The reviewer raises a very interesting point that we had not considered. We now do include the statement that citrulline reactivity per se is not a prerequisite for Th17 responses, using the psoriasis example (page 15). However, discussing the effectiveness of anti-IL-17 therapy in relation to ACPA status falls beyond the scope (and word-limit) of this paper. Variability in anti-IL-17 therapy between complex diseases may be due to numerous factors, including disease heterogeneity, tissue microenvironment and target autoantigen(s). Moreover, psoriasis is now thought to be an innate immune disorder, and it is not clear whether anti-IL-17 is successful because it inhibits Th17 cells, or whether inhibition of innate IL-17 sources (mast cells, neutrophils, γδ T-cells) is more important.

Interestingly, when performing further literature searches we found a recent study that ACPA positivity does correlate with the presence of Th17.1 cells (i.e. IL-17/IFN-g double producers) and Th22 cells, providing support for our finding that citrullination can enhance Th17-cytokines. We now mention this study in the Discussion (page 16).

Response to detailed comments:

‘Page 2 line25, abstract - “The peptide citrullinated on residue 93 (R93Cit) significantly enhanced Th17 development“. The authors have chosen a ‘biased system’ and should mention that pro-IL-17 cytokines are also necessary.’

We now mention the need for pro-Th17 cytokines in the Abstract

‘Page 5 line 26
“Response with slower proliferation kinetics as compared to the native peptide p89-103”
At day 5 and 7, no difference in proliferation is observed between p89-103 and R93 Cit. Could you comment on that and also why 5 days were used for the following experiments (Figure 2 for example)?’

We now point out that proliferation levels are similar at the later time points (page 5). This may reflect that native peptide-stimulated T cells reached the lag phase of the proliferative response quicker (day 3), whilst the R93Cit peptide induced slower proliferation kinetics and reached the lag phase on day 5 – but proliferative rates during the lag phase are similar. We chose day 5 for the re-stimulation experiments shown in Figure 2 and beyond, because by day 5 both the native and R93Cit cultures had reached the lag phase of the proliferative response, so were more comparable than at day 3, when the R93Cit cultures were still in the exponential phase.

‘Page 6 line 17
“re-stimulated with fresh BMDC and a fixed dose of native peptide”
Could the authors explain why the native peptide was used for re-stimulation?’

We used native peptide for the re-stimulation experiments in order to provide an optimal stimulation condition that was standardised across all cultures to justify a strict comparison. In this way, any difference in cytokine production levels in secondary cultures could be attributed to different priming conditions in the primary cultures. Moreover, this protocol is in keeping with that used by previous studies (e.g. Hosken et al, JEM, 1995; Constant et al, JEM, 1995) demonstrating the effect of antigen dose during priming on Th1/Th2 polarisation – they varied the antigen dose during priming but used a single, optimal dose for the secondary cultures.

‘Figure 1A versus 1 B

In Figure 1 A, at 72 hours, proliferation value for p89-10 is around 100. In Figure 1B, at 72 hours, proliferation value for p89-10 is around 60. Could you comment on that difference?'

The data in Fig1 A and B are derived from different sets of experiments. Thus, A shows data from a set of 3 independent experiments and B shows data from another set of 3 independent experiments. We have now made this clear in the Figure legend. The overall level of proliferation was different between the two sets of experiments, for unknown reasons, but could have been caused by, for example, variations in the quality of the BMDC. Nevertheless, relative differences between the native and R93Cit peptides are clear and similar between the different experiments.

'Figure 2A

- Could the control peptide be represented as a dotted line instead of one single point?
- Even low concentration of R93Cit induces production of IFNg as compared to p89-103 (with or W/O Pro-Th17 cytokines). Please comment and discuss this aspect in your manuscript? Specially I would moderate the sentence in discussion page 11 line 21" In contrast, the IFN-γ response was only marginally affected"'

- We have now inserted a horizontal line to indicate the control F1p3 peptide-induced cytokine level.
- We have now moderated the sentence on page 11, stating that the R93Cit peptide supports IFNg secretion at even very low peptide doses. Overall the data suggest that both peptide dose and peptide affinity are important and independent players in determining the outcome of cytokine polarization; this notion is discussed on page 13, 1st paragraph.

'Figure 2B

- Please include a representative flow-cytometry plot (dot blot) for each of the cytokine tested (Can be included in supplementary).
- Please change the y-axis (% of cytokine producing cells) or include abbreviations in the legend.
- no increase in the % of IFNg secreting cells are observed when increasing the peptide concentration of p89-103 as compared to cytokine secretion (Figure 1A). Could you please comment this technical aspect?'

- We now show a representative flow cytometry plot for each of the cytokines tested in Supplementary Figure 1, along with the gating strategy.
- We have modified the Figure 2B y-axis to '% cytokine producing cells'
- The level of cytokine secreted and the number of cytokine-producing T cells are separate measurements than do not necessarily correlate with each other. Theoretically, it could be possible that a certain condition leads to a rise in the number of a cytokine-producing T cell that all produce a lower level

European Journal of Immunology

of that cytokine – thus leading to a reduction in the overall cytokine secretion level in the population. Our data show that citrullination of the p89-103 peptide has a clear and profound effect on Th17 and Th2 responses; both in terms of the level of cytokine secretion and the number of cytokine-producing T cells. As noted by the reviewer, the data seem less clear-cut and variable for IFNg – hence we did not make a big point out of these data, but show them for completeness.

'Figure 3A - Could you include description of peptide control F1p3 in the legend?'

An explanation of the F1p3 control peptide is now included in the legend of Figure 3.

'Figure 3 - Can you change the y-axis (% of RORgT producing cells) or explain abbreviation in the legend?'

An explanation of the y-axis abbreviation RORYT +ve is now given in the legend of Figure 3.

'Figure 5B - Please provide a representative histogram plot (supplementary).'

Representative histogram plots for CD25 are now shown in Supplementary Figure 2.

'Figure 5C. Have you also tested additional pro-Th17 cytokines (GM-CSF and Il-22)?'

No, we did not check other Th17 cytokines, we just focused on IL-17.

'Figure 6: include a representative histogram plot for pSTAT3 and STAT5 at day 0 and 24 with p89-103 and R93Cit.'

Representative histogram plots for pSTATs are now shown in Supplementary Figure 3.

Figure A is shown on the next page.

European Journal of Immunology

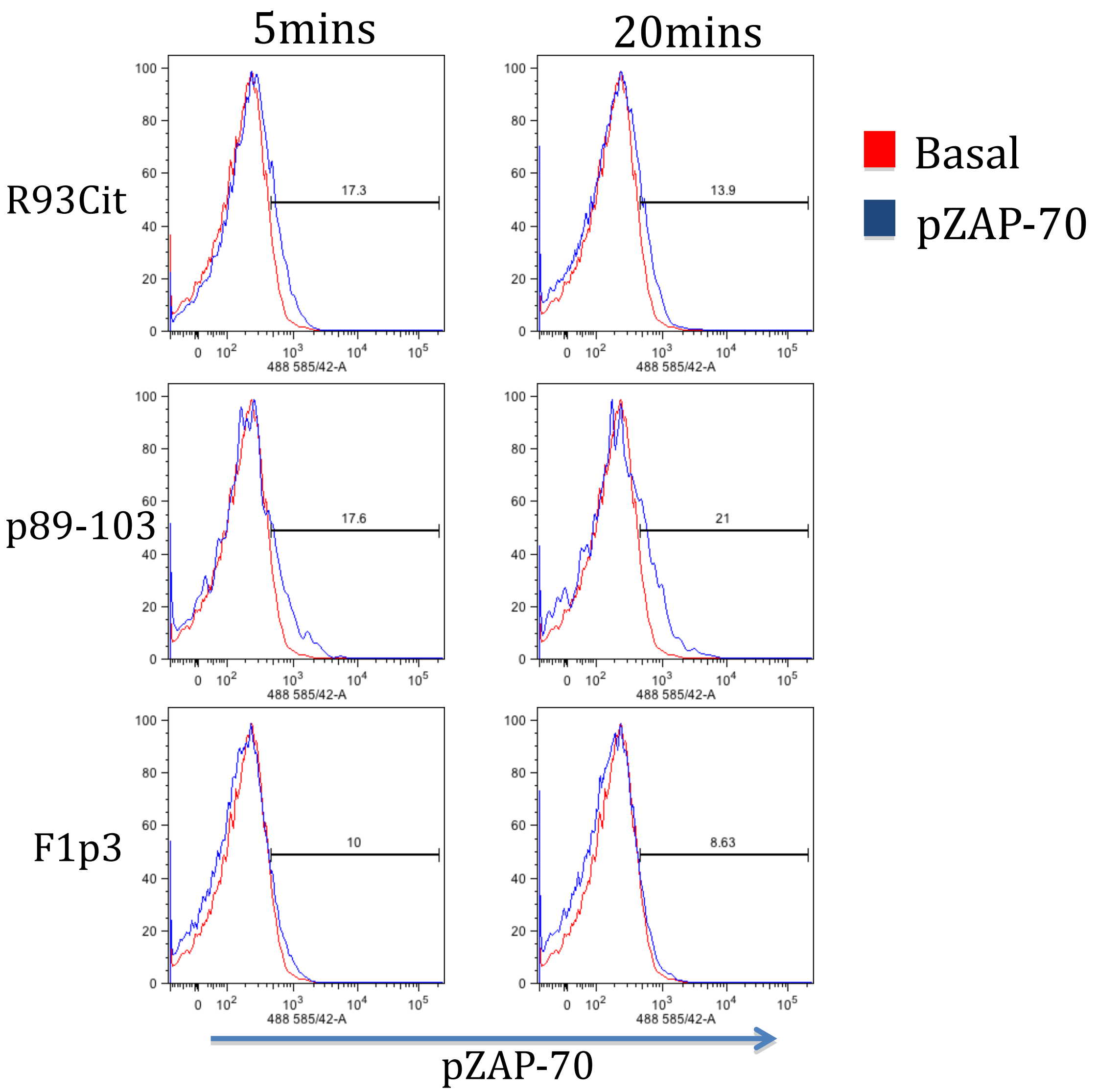


**Second Editorial Decision**

**15-Apr-2016**

Dear Dr. Hilkens,

It is a pleasure to provisionally accept your manuscript entitled "Reduced TCR-dependent activation intensity through citrullination of a T-cell epitope enhances Th17 development by disruption of the STAT3/5 balance" for publication in the European Journal of Immunology. For final acceptance, please

follow the instructions below and return the requested items as soon as possible as we cannot process your manuscript further until all items listed below are dealt with.

Please note that EJI articles are now published online a few days after final acceptance (see Accepted Articles: http://onlinelibrary.wiley.com/journal/10.1002/(ISSN)1521-4141/accepted). The files used for the Accepted Articles are the final files and information supplied by you in Manuscript Central. You should therefore check that all the information (including author names) is correct as changes will NOT be permitted until the proofs stage.

We look forward to hearing from you and thank you for submitting your manuscript to the European Journal of Immunology.

Yours sincerely,
Nadja Bakocevic

on behalf of
Prof. Iain McInnes

Dr. Nadja Bakocevic
Editorial Office
European Journal of Immunology
e-mail: ejied@wiley.com
www.eji-journal.eu_
